# Treatment of Puerperal Fever

**Published:** 1884-02

**Authors:** 


### Treatment of Puerperal Fever.

Dr. T. More Madden (*British Medical Journal*): Whatever other treatment may be required, the employment twice a day of warm antiseptic intra-uterine and vaginal injections, or still preferably of similar irrigations should never be omitted. The use of these to wash out thoroughly all septic matter from the cavity of the uterus is self-evident. At the same time, it is necessary to impress on the nurse the necessity of using the ordinary vaginal syringe with far more caution than is generally observed by ordinary midwives, so as to avoid the risk of injecting air into the open uterine sinuses, or forcing the fluid through the patulous Fallopian tubes into the peritoneal cavity. The precise antiseptic solution used in this way matters comparatively little so that it accomplishes its object of washing away all vitiated exudations or septic matter, and bringing about a healthier condition of the uterine walls and vessels.

The author had used, with almost equal advantage, weak solutions of carbolic acid, permanganate of potash, turpentine, tincture of iodine, sanitas and terebene; and when none of these were at hand, had found an excellent substitute in simple chamomile tea. Care should be taken not to employ over-strong antiseptic intra-uterine injections.

[It seems to us better practice to withhold the intra-uterine douche till vaginal irrigation fails to control the high temperature. If the pyrexia does not yield within a few hours to the vaginal douche properly used, it is safe to assume that the uterine cavity is the source of infection. Its frequent and thorough cleansing is then imperative. But the cavity of the puerperal uterus should not be too hastily invaded. If the vaginal douche is capable of harm, intra-uterine injections are doubly so.]—*American Medical Digest.*

